# An Old Comrades' Guild

**Published:** 1920-11-13

**Authors:** 


### An Old Comrades' Guild.

The Old Comrades' Association of the Royaumont and Villers Cotteret units of the Scottish Women's Hospital, usually known as the Royaumont Association, mustered in great force at the commemoration dinner last year, and over eighty are hoping to be present at the second annual dinner on November 26. As there are at least 500 of the Old Comrades, a proposal is to be brought forward on this occasion by their talented Hon. Secretary, Miss Collum, that a practical "Old Comrades' Guild" should be formed, with an address in London, with use of club dining-room and reception rooms and a letter-forwarding agency, for an inclusive annual subscription of 15s. Miss Collum was formerly radiographer at the Royaumont Hospitals, and is now Editor of the *College of Nursing Bulletin*. A bi-annual news gazette is one of the projects suggested to bind members of the proposed Guild together, and all old members of the medical and nursing staff of the units who are unable to be present at the meeting are invited to communicate, before it takes place, with Miss Collum, 7 Henrietta Street, Cavendish Square, W. 1.

